# *Isospora suis* in an Epithelial Cell Culture System – An *In Vitro* Model for Sexual Development in Coccidia

**DOI:** 10.1371/journal.pone.0069797

**Published:** 2013-07-05

**Authors:** Hanna Lucia Worliczek, Bärbel Ruttkowski, Lukas Schwarz, Kirsti Witter, Waltraud Tschulenk, Anja Joachim

**Affiliations:** 1 Institute of Parasitology, Department of Pathobiology, University of Veterinary Medicine Vienna, Vienna, Austria; 2 Institute of Anatomy, Histology and Embryology, Department of Pathobiology, University of Veterinary Medicine Vienna, Vienna, Austria; University of California, Riverside, United States of America

## Abstract

Coccidian parasites are of major importance in animal production, public health and food safety. The most frequently used representative in basic research on this group is *Toxoplasma gondii*. Although this parasite is well investigated there is no adequate *in vitro* model for its sexual development available and knowledge on this important life cycle phase is therefore scarce. The use of *Isospora* *suis* , a sister taxon to *T. gondii* and the causative agent of piglet coccidiosis, could provide a solution for this. In the present study an *in vitro* model for neonatal porcine coccidiosis in cells representative for the *in vivo* situation in the piglet gut was developed and evaluated. The parasite development was investigated by light and transmission electron microscopy and optimum culture conditions were evaluated. Intestinal porcine epithelial cells (IPEC-J2) adequately representing the natural host cells supported the development of all endogenous life cycle stages of *I* *. suis* , including gametocytes and oocysts. A concentration of 5% fetal calf serum in the culture medium led to highest gametocyte densities on day 12 post infection. Low infection doses (≤1 sporozoite for 100 host cells) were best for oocyst and gametocyte development. The presented system can also be used for immunostaining with established antibodies developed against *T. gondii* (in our case, anti-*Tg*IMC3 antibodies directed against the inner membrane complex 3). The complete life cycle of *I* *. suis* in a cell line representing the natural host cell type and species provides a unique model among coccidian parasites and can be used to address a wide range of topics, especially with regard to the sexual development of coccidia.

## Introduction

Apicomplexan parasites are the causative agents of a wide range of important diseases in humans as well as in animals. Particularly coccidial infections are responsible for significant losses in animal production worldwide and are – in the case of *Toxoplasma gondii* – a major issue in public health and food safety [1]. *T. gondii* is also a well described model organism for research on apicomplexan parasites focusing on cell biology, pathogenesis and host susceptibility or immunity. The methods for research in these areas are well developed and the asexual developmental stages of *T. gondii* can be investigated and manipulated easily in established *in vitro* systems using a variety of host cells [2,3]. As with *Eimeria* species –the most important apicomplexan pathogens in poultry – biotechnological techniques such as transgenic parasites are standard [4–6]. However, no cell culture system is available for many apicomplexan parasites (e.g. *T. gondii*, *Eimeria*, *Cyclospora* , etc.) that allow for studying the sexual and oocyst stages of development. Consequently, an important part of the life cycle of coccidian parasites is so far underrepresented in current research. The understanding of sexual development in *T. gondii* and other coccidia and the development of *in vitro* models for sexual development were indicated as major goals for future research in this field [2,3,7] but to date no system is available to support the routine propagation of coccidial gamogonic stages *in vitro* adequately.

In this work an *in vitro* culture technique supporting all life cycle stages of the porcine coccidium *Isospora* *suis* is described. *I* *. suis* , a sister taxon to *T. gondii* and *Neospora* *caninum* [8], undergoes a direct life cycle and is restricted to the epithelial cells of the intestine of pigs with the highest parasite density in the mid-jejunum. As the causative agent of neonatal porcine coccidiosis, *I* *. suis* leads to an extensive destruction of the epithelial lining and heavy non-hemorrhagic diarrhea in piglets [9,10] and is responsible for significant losses in pig production worldwide. The asexual developmental stages are not classified in generations as for *Eimeria* species but separated in types (type I, type II and subtype II meronts/merozoites) [9]. The first *in vitro* culture of *I* *. suis* was described by Fayer and co-workers in 1984 [11]. In their studies embryonic bovine trachea cells, Madin-Darby bovine kidney cells, porcine kidney cells and bovine colon cells were used as host cells. In all cell types endodyogeny and pairs of merozoites were detected but no further development was observed. In the same year the complete development of *I* *. suis* in the chorioallantoic membrane of chicken embryos was reported by Lindsay and co-workers including a detection of all developmental stages. However, the produced oocysts were not able to sporulate, most probably due to improper environmental conditions necessary for sporulation such as the oxygen level [12]. The evaluation of primary porcine and bovine kidney cells as suitable host cells showed a development of *I* *. suis* until the stage of type II meronts but no gamonts and oocysts were observed [13]. The ultrastructure of developing stages in these cells was described later [14]. The latest attempt on an *in vitro* culture of *I* *. suis* was published in 1998. A swine testicle cell line (ST) was used for propagating the parasites. This system provided complete development to oocysts but no sporulation [15]. Beside these findings *in vivo* infection models for neonatal porcine coccidiosis are well described [16,17] and successfully used for drug efficacy testing [18,19], investigations of the immune response [20–22], and for co-infection modeling [23]. Therefore, a reproducible *in vitro* system including sexual stages and sporulated oocysts together with the knowledge obtained during *in vivo* trials could deliver an integral approach to the understanding of neonatal porcine coccidiosis and coccidian development as well as host-parasite interactions in these parasites.

The aim of the present study was to develop an *in vitro* model for neonatal porcine coccidiosis in cells representative for the *in vivo* situation in the piglet gut. To address this we used the intestinal porcine epithelial cell line IPEC-J2 [24,25] and evaluated optimum culture conditions for successful *in vitro* infections with sporozoites of *I* *. suis* . All developmental stages including macro- and microgametes as well as oocysts were detected reproducibly. Harvested oocysts were able to sporulate only occasionally. The dynamics of *in vitro* development were examined microscopically to determine optimum time points for the investigation of particular stages. Furthermore, it was demonstrated that this system is in principle applicable for the standard technique of immunostaining by using an antibody against a *T. gondii* inner membrane complex (IMC3), a member of a family of proteins which are widely used as markers for cell division dynamics [26]. To the authors’ knowledge the expression of an IMC protein was demonstrated in coccidian microgamont stages for the first time.

The complete life cycle of *I* *. suis* in a cell line representing the natural host cell type and species provides a unique model among coccidian parasites and can be used to address a wide range of topics, especially with regard to the sexual development of coccidia.

## Results

### DEVELOPMENT DEPENDS ON SERUM CONCENTRATION IN THE CULTURE MEDIUM

The optimum concentration of fetal calf serum (FCS) was evaluated for four different infection doses (sporozoites: cells = 1:10, 1:100, 1:200, 1:400) using medium with either 1.25% or 5% FCS. Parasite development and host cell condition were assessed semi-quantitatively [host cells: 0 (no cells left) -3 (cell monolayer intact); parasites: 0 (no stages) -3 (many per field of vision)].

The higher FCS concentration led to a higher density of free and intracellular merozoites (Figure S1). For gametocyte and oocyst development a concentration of 5% FCS also led to a better performance, especially on days post infection (dpi) 12 and 15 (Figure S2). The cell condition was significantly influenced by the FCS concentration for an infection dose of 1:10 on dpi 15; cells cultured in medium with 5% FCS recovered significantly better after the cell destruction due to *the* *I* *. suis* infection than cells in 1.25% medium with regard to confluency of the monolayer (Figure S2).

### THE INFECTION DOSE INFLUENCES THE PRESENCE OF DIFFERENT STAGES AT DIFFERENT TIME POINTS

**Figure 1:**
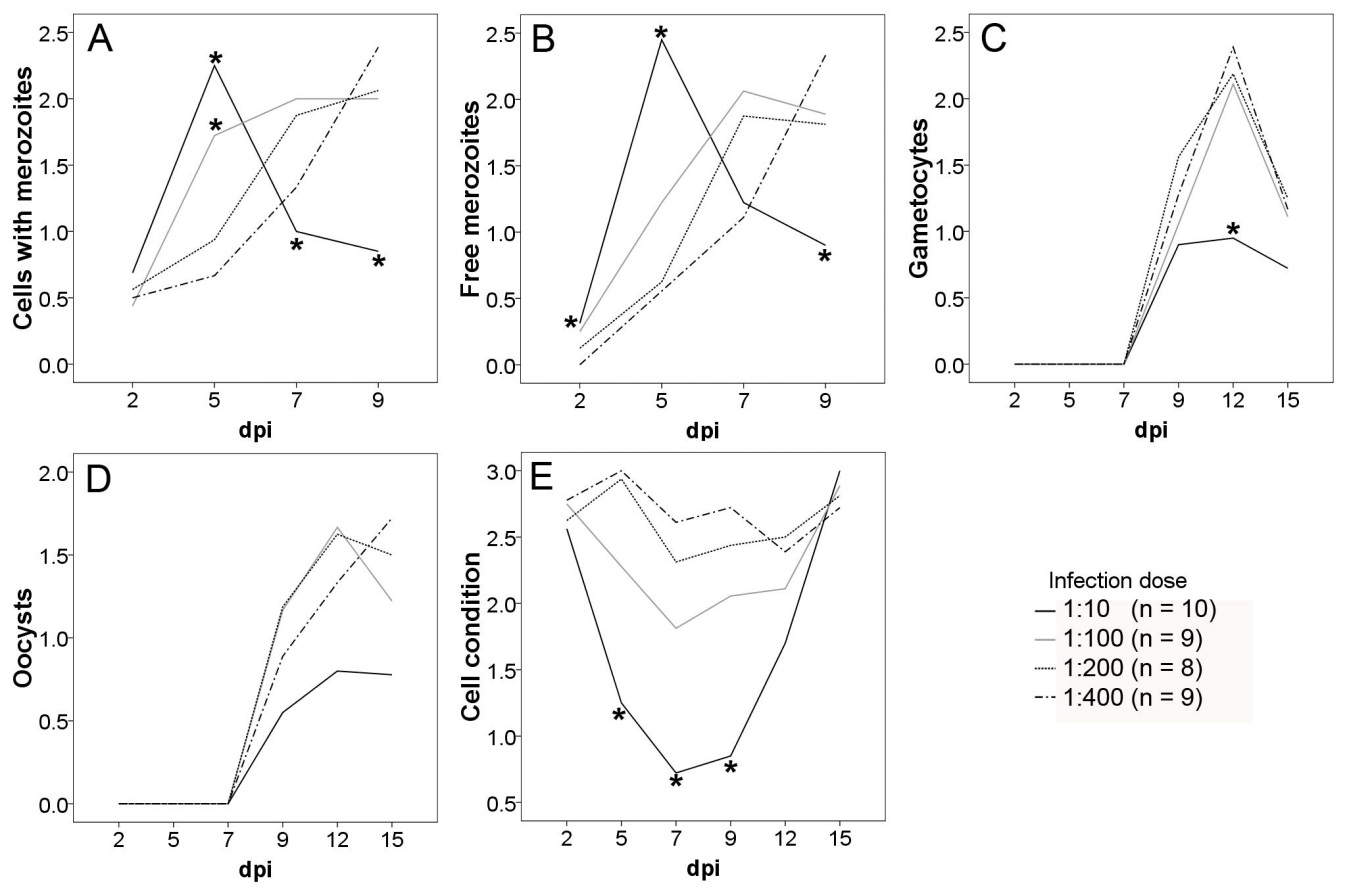
Development of *I* *. suis* stages and cell condition over time dependent on infection dose (sporozoites: cells). Cells were cultivated in medium with 5% fetal calf serum. Group means from semi-quantitative evaluation of developmental stages (A–D) and of host cell condition (E) are shown; *indicates significant difference to at least one other infection dose in multiple comparisons with Bonferroni correction (for details see Table 1); dpi, days post infection. No significant differences were detected for oocysts in multiple comparisons.

The influence of the infection dose (sporozoites: cells = 1:10, 1:100, 1:200, 1:400) on parasite development and host cell condition was analyzed for the optimum FCS concentration in culture medium of 5% in detail (Figure 1). A significant influence of the infection dose on the development of intracellular merozoites (Table S1) was found on dpi 5, dpi 7, and dpi 9. A dose of 1:10 led to the highest density of merozoites on dpi 5, followed by 1:100. On dpi 7 and 9 the situation was inverted, a dose of 1:10 led to the lowest density of merozoites at these time points (Figure 1A Table 1). The density of extracellular merozoites (Table S1) was significantly influenced by the infection dose on dpi 2, 5, and 9. On dpi 5 there were significantly more free merozoites present in the 1:10 infections than in all other doses, on dpi 2 this difference was just found in comparison to 1:400. Later on the density of free merozoites decreased strongly for 1:10 infections, leading to a significantly lower density on dpi 9 (Figure 1B Table 1). The infection dose influenced the number of intracellular gametocytes on dpi 12 (Table S1). They first appeared on dpi 9 in numbers detectable by semi-quantitative analysis, and peaked on dpi 12 for 1:100, 1:200, and 1:400 infections, whereas 1:10 showed a significantly reduced density of gametocytes (Figure 1C Table 1). A possible dependence of oocyst development (Table S1) on infection doses was detected on dpi 12 and 15. In multiple comparisons no significant differences were found, but the mean values showed a tendency towards more oocysts at doses of 1:100 and 1:200 (Figure 1D). The condition score of host cells reflecting the integrity of the cell monolayer (Table S1) was dependent on the infection dose on dpi 5, 7, 9, and 12. For dpi 12 this influence could not be further identified by multiple comparisons. The strongest influence was found for an infection dose of 1:10 leading to the most prominent destruction of the cell monolayer. On dpi 5, 7, and 9 the cell condition was significantly worse for this infection dose, but a recovery of the cell monolayer was observed until dpi 15 (Figure 1E Table 1).

**Table 1:** Significant differences between infection doses for parasite development and cell condition over time in culture medium with 5% FCS.

	**dpi**	**Infection**	***n***	**Mean (SD)**	**Significant**	***n***	**Mean (SD)**	***p*-value**
		**dose**			**difference to**			
**Cells with**	5	1:10	10	2.25 (0.42)	1:100	9	1.72 (0.67)	0.015
**Merozoites**					1:200	8	0.94 (0.32)	< 0.001
					1:400	9	0.67 (0.25)	< 0.001
		1:100	9	1.72 (0.67)	1:200	8	0.94 (0.32)	0.001
					1:400	9	0.67 (0.25)	< 0.001
	7	1:10	9	1.00 (0.71)	1:100	8	2.00 (0.53)	0.035
	9	1:10	10	0.85 (0.34)	1:100	9	2.00 (0.83)	0.007
					1:200	8	2.06 (0.99)	0.005
					1:400	9	2.39 (0.55)	<0.001
**Free**	2	1:10	8	0.31 (0.26)	1:400	9	0.00 (0.00)	0.043
**Merozoites**	5	1:10	10	2.45 (0.37)	1:100	9	1.22 (0.71)	0.001^§^
					1:200	8	0.63 (0.23)	< 0.001^§^
					1:400	9	0.56 (0.17)	< 0.001^§^
	9	1:10	10	0.90 (0.81)	1:400	9	2.33 (0.56)	0.003
**Gametocytes**	12	1:10	10	0.95 (0.76)	1:100	9	2.11 (0.78)	0.005
					1:200	8	2.19 (0.53)	0.004
					1:400	9	2.39 (0.60)	< 0.001
**Cell condition**	5	1:10	10	1.25 (0.63)	1:200	8	2.94 (0.18)	< 0.001^§^
					1:400	9	3.00 (0.00)	< 0.001^§^
	7	1:10	9	0.72 (0.51)	1:100	8	1.81 (0.70)	0.004
					1:200	8	2.31 (0.59)	< 0.001
					1:400	9	2.61 (0.55)	< 0.001
	9	1:10	10	0.85 (0.63)	1:100	9	2.06 (0.85)	0.002
					1:200	8	2.44 (0.56)	< 0.001
					1:400	9	2.72 (0.51)	< 0.001

*P*-values from post-hoc multiple comparisons with Bonferroni corrections are shown (significance assumed for *p* ≤ 0.05); ^§^ indicate results from multiple Mann-Whitney (Wilcoxon) two-sample test with Bonferroni correction for not normally distributed data (significance assumed for *p* ≤ 0.008); only results with significant differences are shown; post-hoc tests were performed for data indicating an influence of the infection dose in ANOVA or Kruskal-Wallis tests. Infection doses are given as a ratio of sporozoites: cells. Parasitic stages: 0 (negative) to 3 (many/field of vision); cell condition: 0 (no cells left) to 3 (monolayer). No significant differences were detected for oocysts in multiple comparisons; dpi: days post infection.

### *I* *. SUIS* SHOWS LOW INFECTION RATES WITH A MAXIMUM ON CULTURE DAY 4

**Figure 2:**
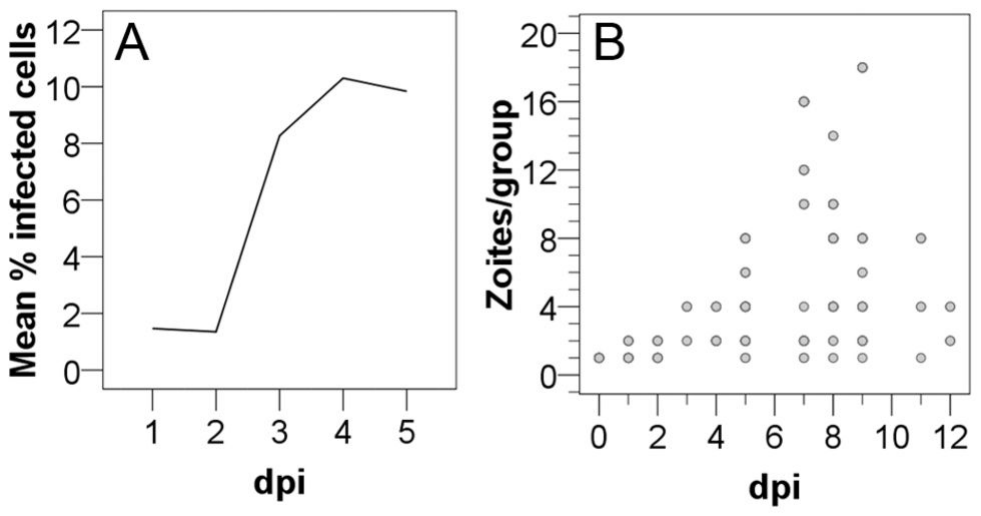
Dynamics of *I* *. suis* in intestinal porcine epithelial cells (IPEC-J2). Cells were infected with an infection dose of 1:10 (sporozoites: cells), medium with 5% FCS was used. The percentage of infected cells over time (A) and the number of zoites per group over time (B) are shown. A group was defined as the totality of merozoites derived from one dividing mother cell based on the arrangement of zoites in pairs or rosettes; dpi, days post infection.

Infection rates for all tested conditions were low compared to *in vitro* infection rates of other apicomplexan parasites. For an infection dose of 1:5 (sporozoites: cells) an average of 1.90% (SD 0.57; n = 4) of cells were initially infected. For 1:10 infections 1.46% (SD 0.37; n = 5), for 1:50 infections 0.18% (SD 0.01, n = 2), and for 1:100 infections 0.19% (SD 0.13, n = 9) of cells were infected with sporozoites. Higher infection doses than 1:5 did not lead to higher infection rates (data not shown). For a dose of 1:10 quantitative kinetics of intracellular merozoites were evaluated until dpi 5. On dpi 3 parasite spreading across the culture was quantifiable (8.27% infected cells), reaching maximum infection rates on dpi 4 (10.3% infected cells; Figure 2A). The number of zoites per group increased over time, reflecting the start of merogony. The maximum group size (n=18 parasite cells/group) was seen on dpi 9 (Figure 2B). A group was defined as the totality of merozoites derived from one dividing mother cell based on the arrangement of zoites in pairs or rosettes within one parasitophorous vacuole (PV).

### ALL DEVELOPMENTAL STAGES WERE OBSERVED *IN VITRO*

**Figure 3:**
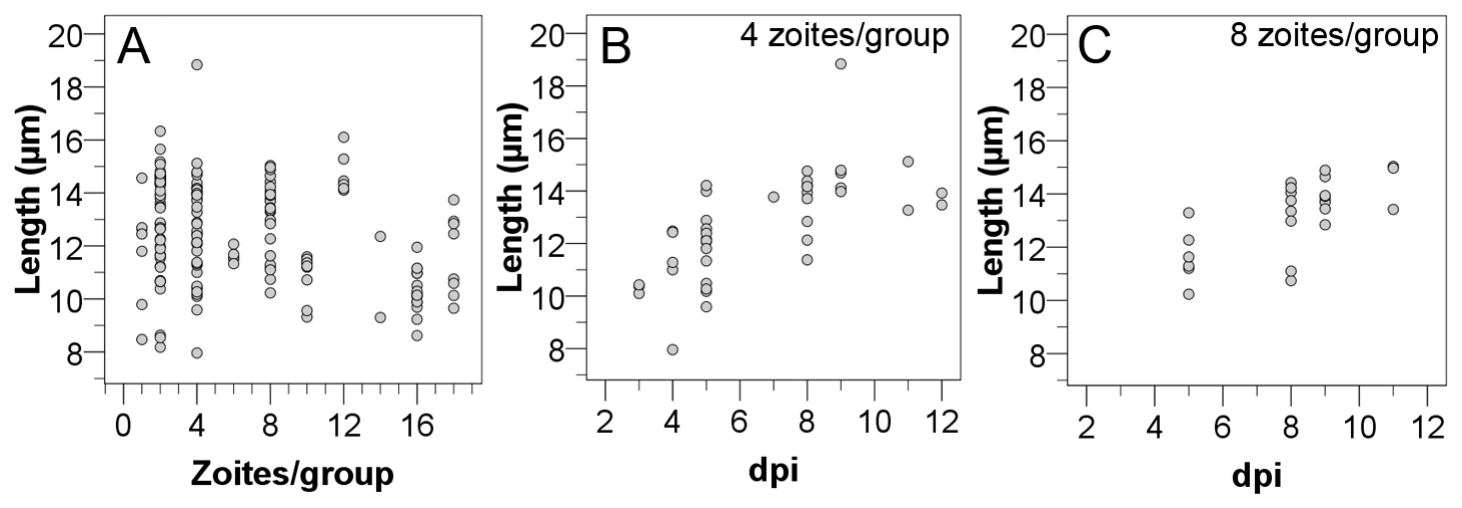
Size of intracellular *I* *. suis* zoites in intestinal porcine epithelial cells (IPEC-J2). The correlation between length and group size (A) and the correlation between length and dpi for groups of 4 zoites (B) and 8 zoites (C) are shown. Groups of 4 zoites were first detected at 3 dpi, groups of 8 at 5 dpi. A group was defined as the totality of merozoites derived from one dividing mother zoite based on the arrangement of zoites in pairs or rosettes; dpi, days post infection.

**Figure 4:**
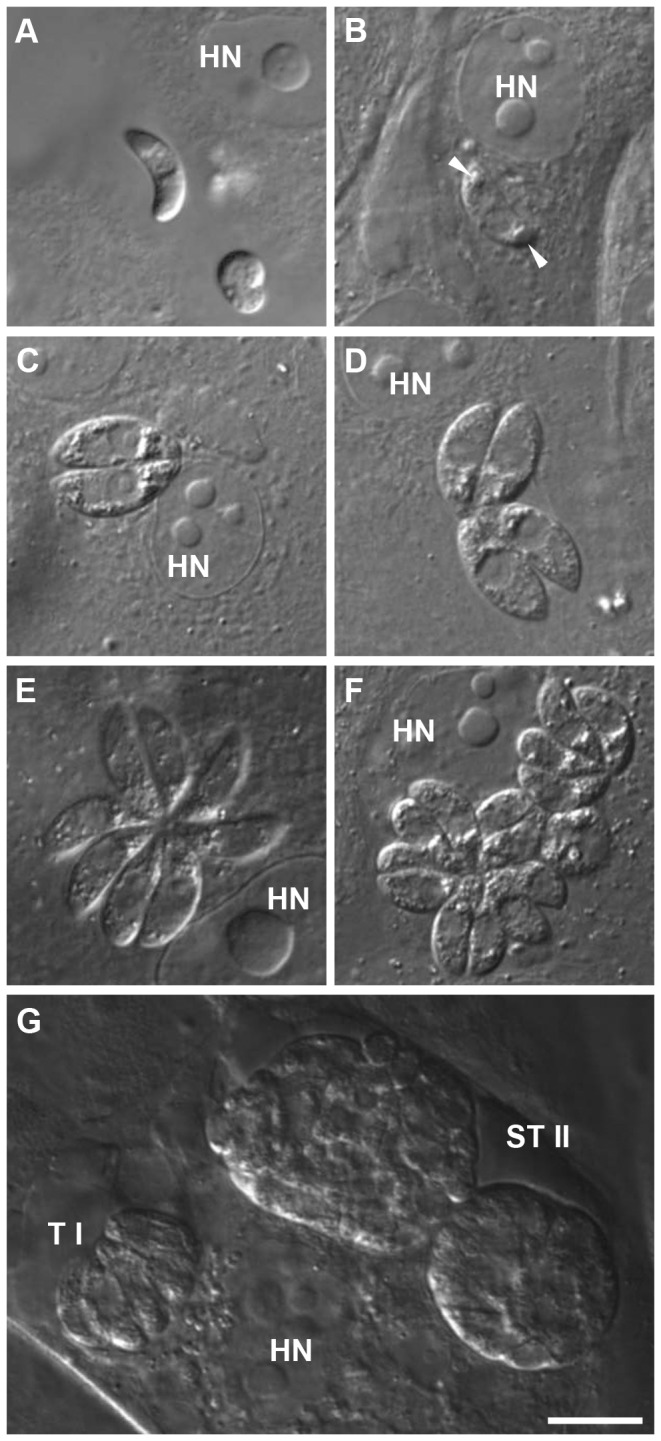
Sporozoites and merozoites of *I* *. suis* *in vitro* in intestinal porcine epithelial cells (IPEC-J2). (A) extracellular sporozoite before cell invasion on dpi 0; (B) intracellular sporozoite 24h after infection, note the anterior and posterior refractile body (arrow heads); (C), (D), and (E) type I merozoites on dpi 5; (F) smaller presumptive type II merozoites on dpi 7; (G) two large groups of subtype II merozoites (ST II) and a small group of type I merozoites (TI) on dpi 9. HN, host cell nucleus; dpi, days post infection; bar = 10 µm.

**Figure 5:**
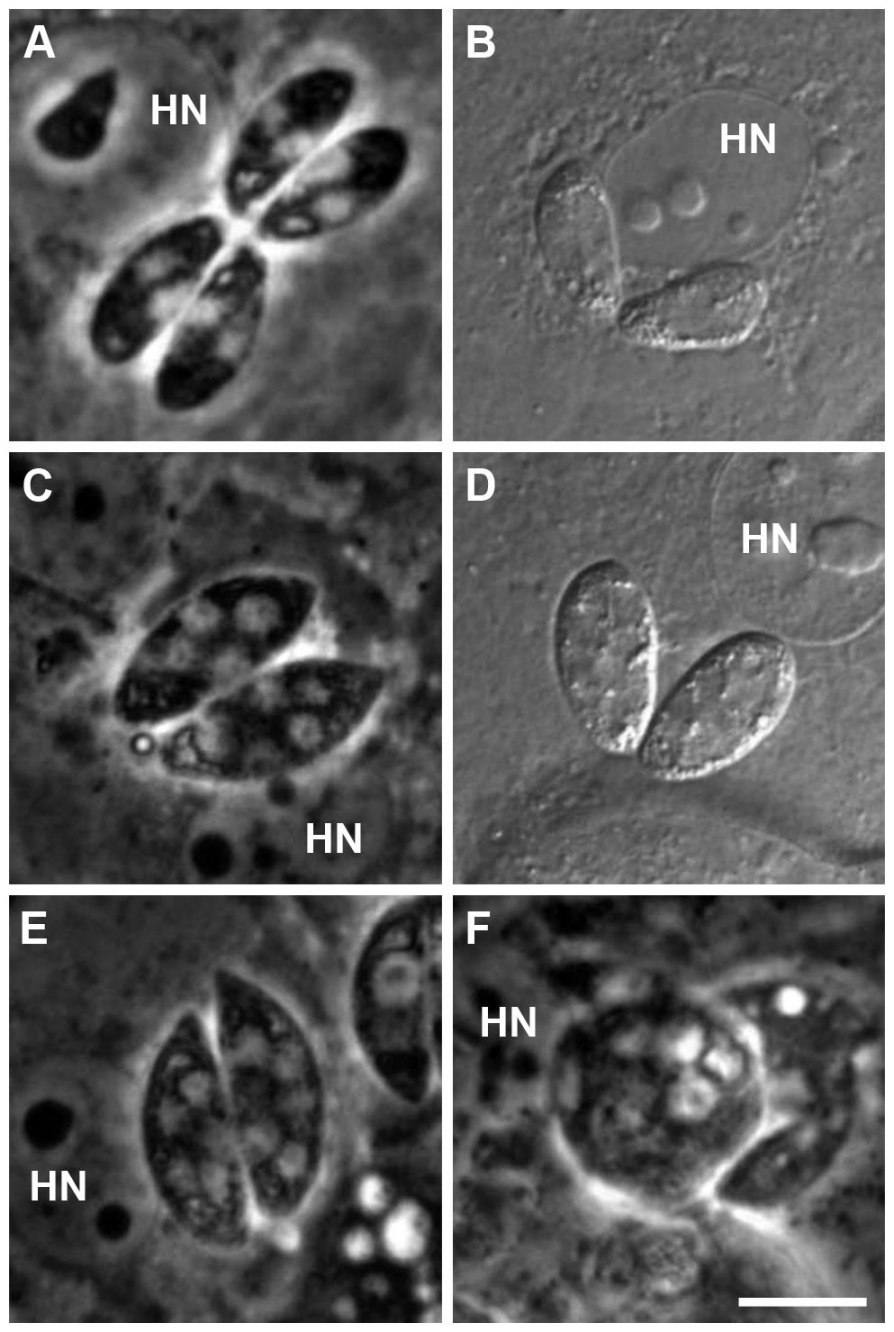
Meronts of *I* *. suis* in the *in vitro* culture in intestinal porcine epithelial cells (IPEC-J2). (A) PC image on dpi 9 and (B) DIC image at dpi 3 of bi-nucleated type I meronts; (C) PC and (D) DIC image of symmetric type II meronts with multiple nuclei; (E) PC image of asymmetric type II meronts on dpi 9; (F) rounded multinucleated subtype II meront on dpi 9. DIC, differential interference contrast; dpi, days post infection; HN, host cell nucleus; PC, phase contrast; bar = 10 µm.

All developmental stages described in the literature for *in vivo* as well as for *in vitro* infections could be observed. However, a classification of intracellular groups of merozoites to types was not unambiguously possible based on zoite length and shape. The length of individual zoites differed between different group sizes. Especially within groups of 2 merozoites (8.2-16.3 µm, mean 13 µm, SD 1.9), 4 merozoites (8.0-18.8 µm, mean 12.7 µm, SD 2.0), and 8 merozoites (10.2-15.0 mm, mean 13.2 µm, SD 1.4) the sizes varied strongly (Figure 3A). When evaluating length of merozoites split by group size and dpi, the variation was smaller and an increase in size was seen over the course of time (Figure 3B, C). Sporozoites were motile during their search for an appropriate host cell, elongated and mostly crescent-shaped (Figure 4A). After invasion of the host cell their shape became blunt and refractile bodies were visible (Figure 4B). Merozoites on dpi 5 assumed to be type I were found in groups of different sizes (Figure 4C–E). Stages assumed to be type II merozoites, because of their smaller size and their shape, were observed from dpi 7 on (Figure 4F). Big groups of small merozoites in a convex PV considerably exceeding the host cell monolayer were assumed to be subtype II merozoites (Figure 4G). A clear classification into types could be made for meronts based on the numbers of nuclei. Binucleated type I (Figure 5A, B) and multinucleated type II meronts (Figure 5C, D) could be observed, as well as rounded multinucleated subtype II meronts (Figure 5F). Type II meronts appeared in two different shapes, symmetric stages (Figure 5C, D) as well as asymmetric ones (Figure 5E). Type I stages were present in the culture before type II stages. Beside this observation there was no strict sequence of development observable; at later time points all types were present simultaneously.

**Figure 6:**
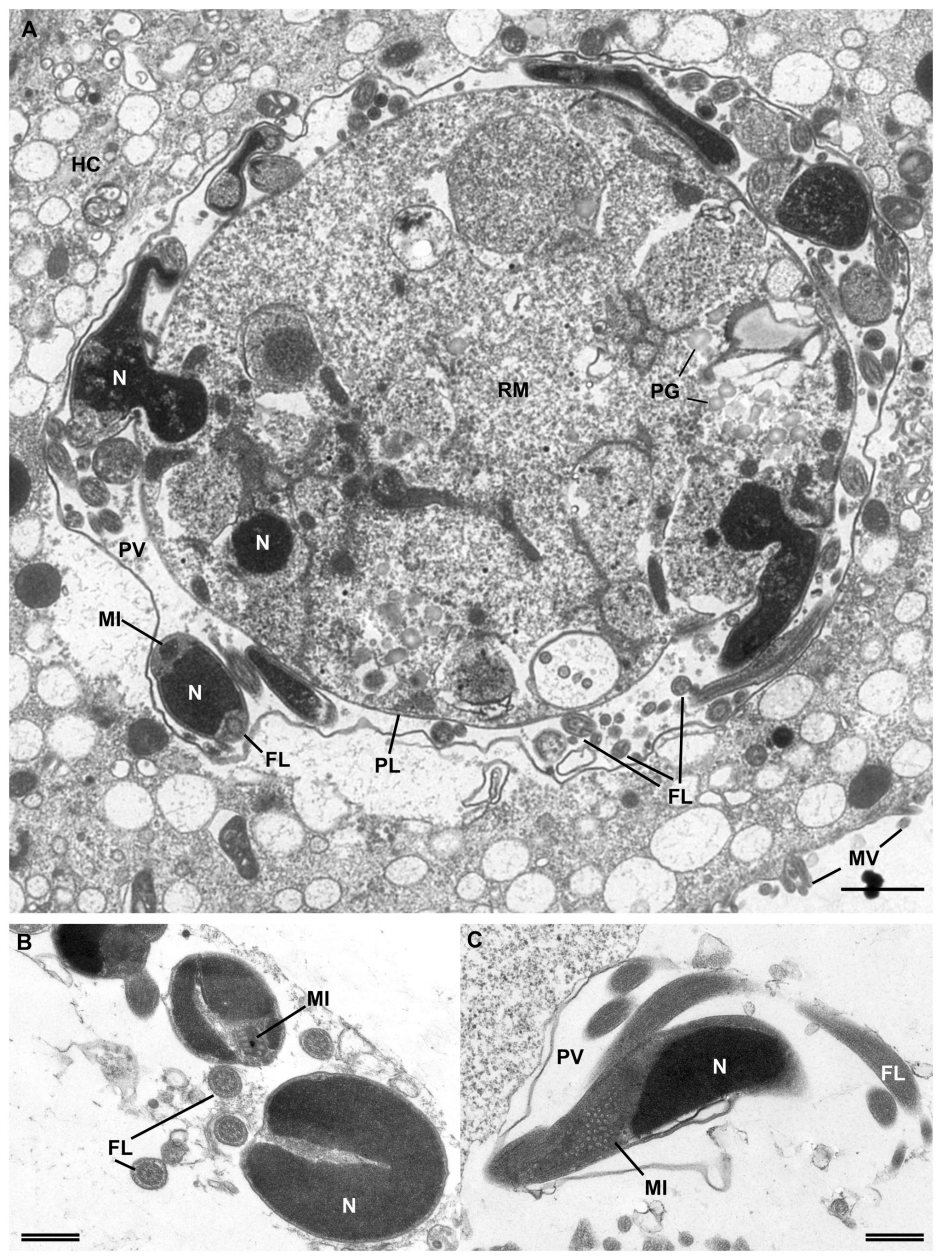
Electron micrographs of a microgamont and microgametes of *I* *. suis* in intestinal porcine epithelial cells (IPEC-J2). (A) section through a microgamont with budding microgametes on dpi 13; (B) transversal section through microgametes and flagella showing the 9x2 + 2 arrangement of microtubules within the flagella on dpi 12; (C) longitudinal section through the flagella and the body of a microgamete showing the typical large mitochondrion in the latter. dpi, days post infection; FL, flagella; HC, host cell; MI, mitochondrion; MV, host cell microvilli; N, nucleus; PG, polysaccharide granules; PL, plasmalemma; PV, parasitophorous vacuole; RM, residual cytoplasmatic mass; single bar = 1000 nm, double bar = 500 nm.

**Figure 7:**
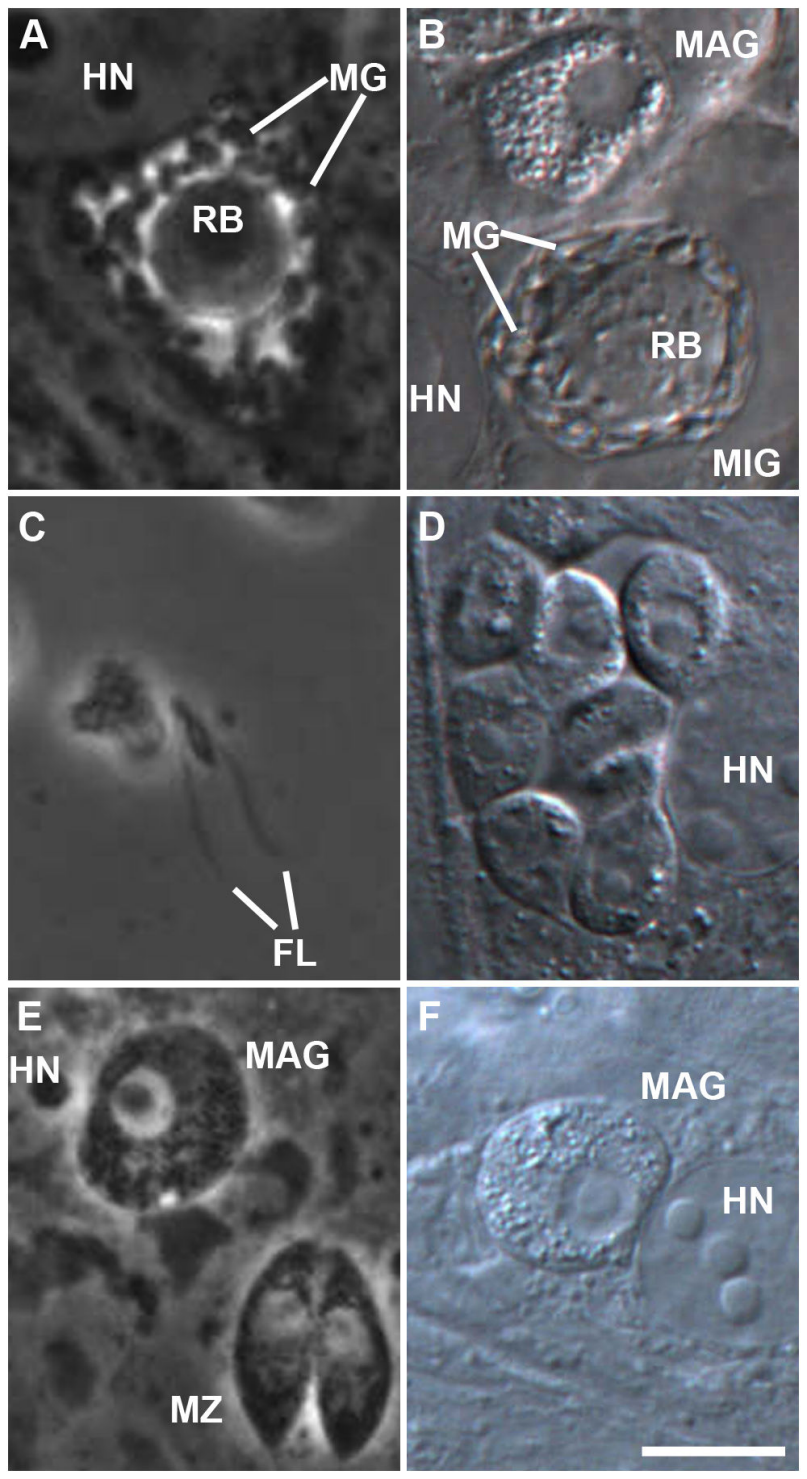
Sexual stages of *I* *. suis* in intestinal porcine epithelial cells (IPEC-J2). (A) PC image of a mature microgamont with intracellular microgametes (MG) surrounding the residual body (RB) on dpi 14; (B) DIC image of a mature microgamont (MIG) and a macrogamete (MAG) in the neighbouring host cell on dpi 13, notice the pronounced granular structure of the MAG; (C) PC image of a free microgamete in motion showing the typical two flagella (FL) on dpi 8; (D) presumptive young macrogamonts within one parasitophorous vacuole, notice the distinct nucleoli and the granular structure of the cytoplasm typical for macrogamonts; (E) PC image of a mature macrogamete and a pair of type I merozoites (MZ) on dpi 7; (F) DIC image of a mature macrogamete on dpi 19. DIC, differential interference contrast; dpi, days post infection; HN, host cell nucleus; PC, phase contrast; bar = 10 µm.

Single immature gametocytes were first detected on dpi 6. Mature micro- and macrogametocytes in higher frequencies were present from dpi 9 onwards. The fine structure of microgametocytes was investigated by electron microscopy (Figure 6) and was found to be similar to these stages in the pig gut [10]. Microgametes were found revolving around a residual body within the PV (Figure 7A, B) or free in the supernatant, exhibiting two flagella (Figure 7C). Some of the structures detected possibly represent young macrogamonts because of the pronounced nucleolus and granularity of the cytoplasm (Figure 7D). Mature macrogamonts showed the same features but were larger in size (Figure 7E, F). Gametocytes were detected in highest densities at the edge of the culture wells.

The used method for excystation led to a mean excystation rate of 63%. Therefore, the infection material contained sporulated oocysts which were not excysted but also non-sporulated oocysts. The non-sporulated oocysts from the inoculum did not attach to the host cells and could therefore be removed with the first medium exchange one day post infection; subsequently, no non-sporulated oocysts were detected intra- or extracellular until dpi 9. The majority of sporulated oocysts contaminating the inoculum were also removed with the first medium exchange. Occasionally, sporulated oocysts from the inoculum were detected attached to or phagocytized by the host cells and were not removable with the medium exchange. Until dpi 9 the contaminating sporulated oocysts were observed to degrade reflected by a granular content instead of sporozoites (Figure S3A) within the sporocysts or collapsed oocysts and sporocyst walls (Figure S3B).

**Figure 8:**
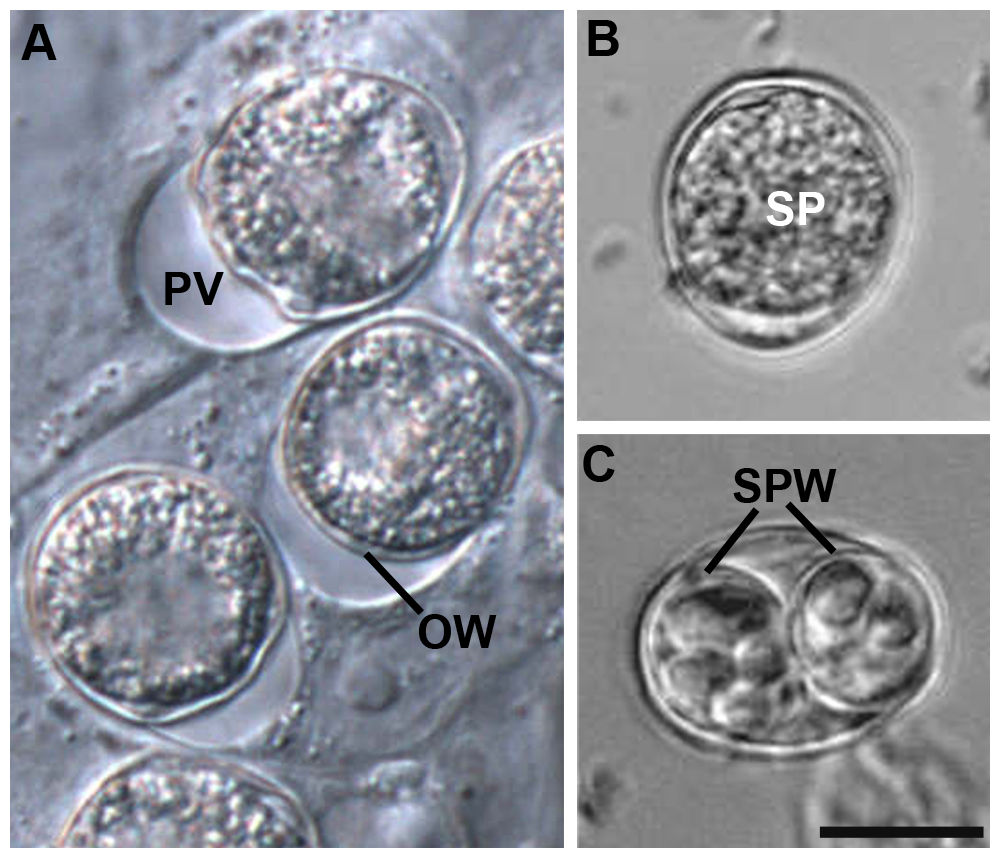
Oocysts of *I* *. suis* in the *in vitro* culture. (A) intracellular oocysts with a distinct parasitophorous vacuole (PV) and an already developed oocysts wall (OW) on dpi 13; (B) free unsporulated oocyst containing the sporont (SP); (C) sporulated oocyst 5 days after harvest from cell culture and incubation in tap water at room temperature with developed sporocyst walls (SPW) containing sporozoites; dpi, days post infection; bar = 10 µm.

Intracellular non-sporulated oocysts developed in the culture were first observed on dpi 9 and were subsequently also found in the supernatant. As for the gametocytes, they were found in highest densities at the edge of the culture wells. They showed a starting contraction of the sporont already within the host cell (Figure 8A). Freshly harvested non-sporulated oocysts were comparable in morphology to oocysts from piglet faeces as investigated by light microscopy (Figure 8B), but most of the oocysts lost this morphology with a contracted sporont during incubation for sporulation, resulting in an oocyst wall with diffuse content. After incubation of the harvested supernatant for 4-7 days recovered sporulated oocysts had the typical morphology with two sporocyst walls containing four sporozoites each (Figure 8C).

### HARVESTED OOCYSTS SPORULATED ONLY OCCASIONALLY BUT WERE INFECTIOUS *IN VIVO*

Non-sporulated oocysts were harvested from cell culture supernatants during various experiments using both serum concentrations and different infection doses. Directly after harvest no sporulated oocysts were detected on any occasion indicating that no non-excysted infectious oocysts from the infection material were contaminating the harvested supernatant. Subsequent sporulation was observed in some cases but could not be reproduced reliably. Altogether, sporulation of harvested oocysts was observed in eight experiments. In seven of them a quantity of 8 x 10^5^ cells were seeded in 25 cm^2^ cell culture flasks. They were infected with a dose of 1:10 (sporozoites: cells; n = 3; corresponding to sporozoites from 10,000 oocysts/per experiment) or 1:40 (n = 4, ^≈^ 2,500 oocysts/per experiment) in medium with 1.25% FCS. On dpi 12 a mean number of 1388 oocysts (SD 807) could be harvested from 1:10 infected flasks with a mean sporulation rate of 1.29% (SD 0.7) and a calculated recovery of 14.9% (SD 8.1) in relation to calculated oocysts used for infection. From 1:40 infected flasks a mean number of 300 oocysts (SD 339) could be harvested on dpi 12 with a mean sporulation rate of 36.1% (SD 17.3) and a calculated mean recovery of 12.0% (SD 13.6).

Additionally, 142 sporulated oocysts could be obtained after incubation of the supernatant harvested from three wells of a 6-well plate infected with a dose of 1:500 in culture medium with 5% FCS. These oocysts were used for the infection of one piglet at the first day of life to demonstrate infectivity of these oocysts. The infected piglet shed a countable number of oocysts on the 7^th^ day post infection with 2.74 x 10^5^ oocysts per gram faeces (opg); shedding dynamics of this piglet were not investigated further.

#### ANTI-*Tg*IMC3 ANTIBODIES BIND TO *I* *. SUIS* STAGES *IN* *VITRO*

**Figure 9:**
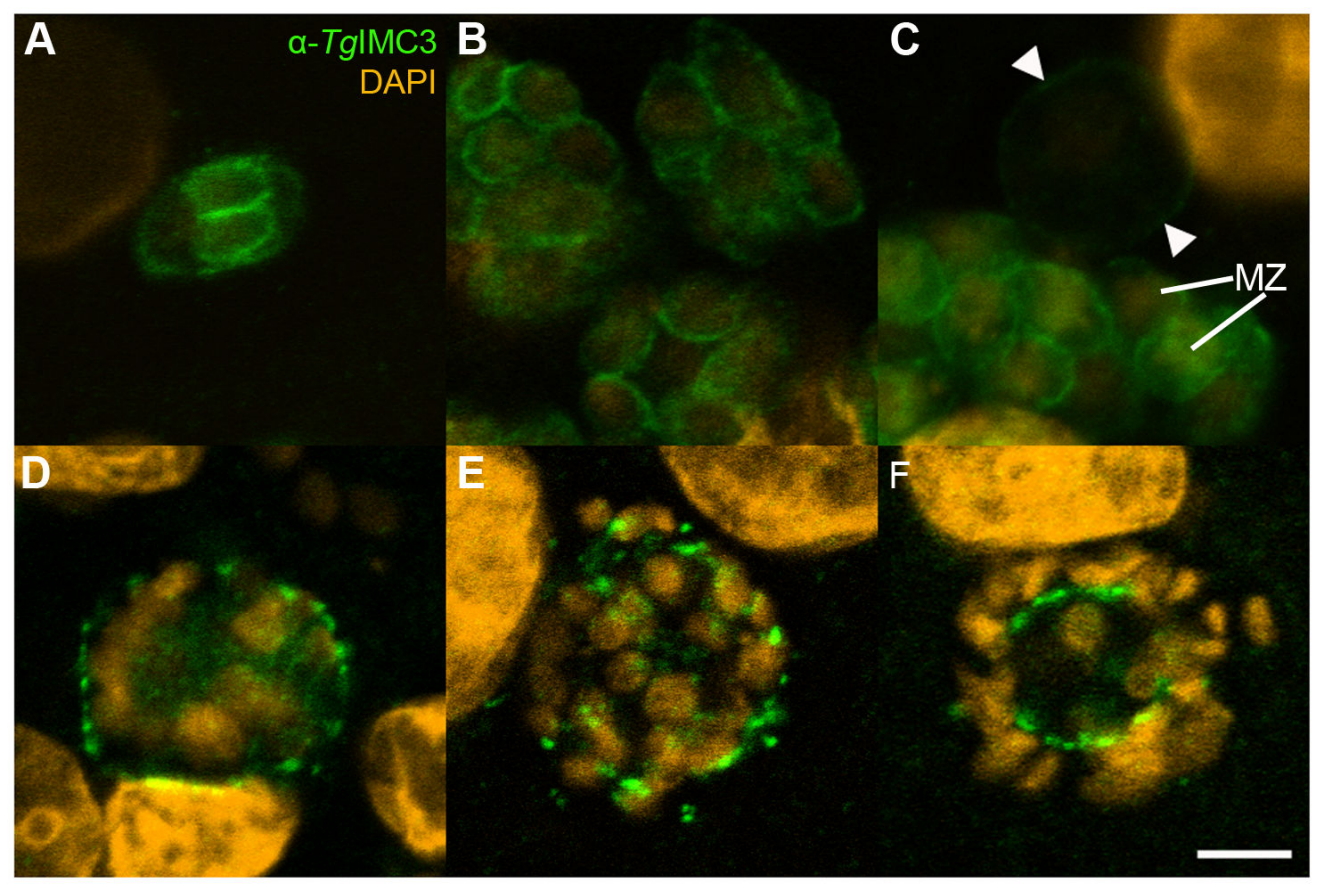
Immunohistochemical staining of *I* *. suis* stages with anti-*Tg*IMC3 and DAPI. Staining with anti-*Tg*IMC3 is shown in green, DAPI-staining is depicted as orange; (A) Type I meront, dpi 8; (B) merozoites, dpi 8; (C) merozoites and a weakly stained macrogamont indicated by arrow heads, dpi 8; (D), (E) presumptive early microgamonts, dpi 11; (F) mature microgamont with microgametes surrounding the residual body, dpi 11; dpi, days post infection; bar = 5 µm.

Expression of IMC3 was found in meronts and merozoites of *I* *. suis* (Figure 9A, B). Developing daughter cells within a mother cell showed a stronger signal than the mother cell itself (Figure 9A). In addition, IMC3 could be detected in sexual stages. In macrogamonts only a weak staining was observed compared to merozoites (Figure 9C). In microgamonts the staining pattern was dependent on the maturation level of the gamont. In presumptive early microgamonts a scattered staining pattern located close to the surface of the gamont was observed (Figure 9D). After further nuclear division (leading to maturation) of the presumptive microgamont, the pattern became more irregular (Figure 9E). In mature microgamonts, when single microgametes were already distinguishable from the residual body, the scattered staining was found central of the gametes, located closely to the surface of the residual body (Figure 9F). In intracellular oocysts no staining with anti-IMC3-antibody was observed (data not shown).

## Discussion

IPEC-J2 cells support the complete endogenous life cycle of *I* *. suis* *in vitro*. This cell line generally provides an excellent *in vitro* system for gut epithelial responses to pathogens; it is well characterized and already in use for investigations on other gut pathogens of juvenile animals [27].

The conditions for cultivation of *I* *. suis* vary according to the targeted developmental stage. An infection dose of 1:10 (sporozoites: cells) appeared to be best for merozoite harvesting on dpi 5. If gametocytes or oocysts are in the focus, doses of 1:100, 1:200, or 1:400 showed the best performance; dpi 12 is the preferred day for harvest or fixation of these stages. A dose of 1:10 led to the most severe cell destruction but host cells recovered until dpi 15. Therefore this setting might be representative for pathological changes in the piglet gut epithelium *in vivo* where the intestinal lining has recovered by dpi 14 [16]. For investigations on host cell response to the parasite the infection dose has to be selected carefully – too much epithelial destruction might lead to a shortage of affected viable host cells. On the other hand at least a mild pathology should be present – these requirements could be fulfilled by choosing an infection dose of 1:100 or slightly higher. For a model focusing on pathological changes in the epithelium the use of polarized cells should be considered. Such systems are well developed for IPEC-J2 [25,28] and should be adaptable for infections with *I* *. suis* . A disadvantage of these systems is the low resolution capacity in light microscopy due to the use of mostly opaque membranes, therefore live microscopy and imaging in parallel with e.g. gene expression studies is difficult [28].

Compared to other coccidia *I* *. suis* showed relatively low infection rates *in vitro* with a maximum of 1.9% infected cells one day post infection. Infection rates depend on parasite strains, host cells and culture conditions, but e.g. for *T. gondii* rates from >15% to 70% have been described [29,30]. For *Eimeria bovis* infection rates >12% were reported [31]. Even the use of higher infection doses did not lead to more cells infected by *I* *. suis* sporozoites. This observation is in good agreement with *in vivo* data where – compared to poultry or rodent coccidia – only a low density of parasitic stages is found in the gut epithelium [9,10]. High infection doses (1:10) lead to severe cell destruction and might consequently prohibit parasite proliferation and high numbers of sexual stages due to the absence of host cells; therefore there is an upper limit to the number of sporozoites that can be used for infection, especially for the investigation of later developmental stages.

Asexual developmental stages of *I* *. suis* are described as types characterized by their shape, length, width, group size, and in case of meronts the number of nuclei [12,13,32]. In this study different types of merozoites were unambiguously present but a clear classification based on measurements was not possible. Therefore the kinetics of asexual development were not investigated further but the dynamics in stage appearance seem to be comparable to *in vivo* studies [9,10].

The development of sexual stages was significantly delayed compared to *in vivo* infections. Infected piglets start oocysts shedding about 4-6 days after infection [9,10,16,33,34], whereas in the present study oocysts were not detected before dpi 9. *In vivo* gamonts are detectable from dpi 4 or 5 on [9,10], in this study young gametocytes were first detected on dpi 6. Surprisingly, the highest density of merozoites was found on dpi 5, at the same time as described for the piglet gut [9]. Therefore it can be assumed that the significant delay in development occurs during gametocyte maturation or oocyst formation. A longer intracellular retention time of oocysts compared to the *in vivo* situation leads to a longer exposure to low oxygen levels and could inhibit oocyst sporulation [12]. This might be one explanation for low sporulation rates or - as in the majority of experiments – the absence of sporulation. Besides, various possible triggers are present in the piglet gut but not in the *in vitro* culture, such as shear stress due to chymus movement. A delayed release of oocysts from host cells could be due to the lack of physiological/pathological sloughing of the gut epithelium. Factors responsible for gametocyte maturation have to be identified to improve the presented *in vitro* system further, first of all by further systematic variation of culture conditions. Regarding the location of developed sexual stages we assume, that higher concentrations of gametocytes near the outer border of the culture vessels could be an effect of the meniscus of the culture medium since peripheral accumulation of sexual stages was not observed in channel slide systems that prevent formation of a meniscus (own, unpublished results). This observed peculiarity could be used to design culture formats with enriched surfaces to support increased gametocyte and oocyst formation. Oocysts harvested *in vitro* were shown to be infective *in vivo*. The infectivity *in vitro* still has to be tested and needs further optimization of the presented culture system as a basis to provide a reproducible output of sporulating and infectious oocyst.

As a first step for further analyses, the application of already existing tools for research on stage development in apicomplexa has been tested. Immunostaining of fixed cells is – beside a variety of more modern techniques – still a widely used tool, especially if no transfection system is established for the organism in question [35]. IMC3 was chosen as a representative of a subfamily of inner membrane complex proteins. Some of these molecules are highly conserved, indicating the possibility of cross reaction of specific anti-*T. gondii*-IMC3 antibodies with corresponding proteins in *I* *. suis* . Staining of IMC molecules is used for research on the cytoskeleton assembly itself and as supporting staining for experiments focusing on other subcellular structures [26]. IMC3 expression was detectable by immunostaining in a similar pattern to *T. gondii* in meronts and merozoites [26]. Moreover, a distinct IMC3 staining was seen in microgamonts of *I* *. suis* . Early microgamonts are addressed as ‘presumptive’ because a distinct marker for this stage was not available for immunostaining. They were solely differentiated from meronts due to their morphology and the difference in IMC3 staining. Differentiation based on changes in the nuclear structure as suggested for *T. gondii* by Ferguson et al [36] would need ultrastructural resolution techniques not applicable in this setting. Staining of the gametic transmembrane protein GCS1, which was shown to be expressed in *Plasmodium* male gametocytes and is assumed to be highly conserved in sexual stages of various organisms [37], could be used in further studies to identify young microgamonts unambiguously. A clear identification of mature microgamonts was possible by light microscopy showing the residual body and surrounding microgametes in a PV. Unlike microgametogenesis described in *Isospora* *felis* (syn. *Cystoisospora* *felis* ) [38], no deep invaginations of the gamont surface appear in *I* *. suis* . The absence of such deep invaginations was previously described by Matuschka and Heydorn [10] for intestinal stages of *I* *. suis* and observed in electron micrographs from *in vitro* culture in this study. An additional difference is the number of microgamonts produced by one mother cell. *I* *. felis* can give rise to several hundred microgametes whereas for *in vivo* development of *I* *. suis* up to 60 microgametes were described [10]. This might reflect a closer analogy of microgametogenesis in *I* *. suis* to *T. gondii* [36,39]. During our study up to 30 microgametes could be identified although no systematic investigation was carried out.

With the present knowledge the authors cannot suggest a defined structure in *I* *. suis* showing the IMC3 expression. It appeared to be expressed adjacent to the cell membrane of the microgamont in light microscopy. In early development the expression appears to be evenly scattered throughout the periphery of the microgamont. After extensive nuclear division the pattern becomes more irregular, suggesting a movement towards the central region of the microgamont or a change in the surface structure due to the start of the budding process. When mature microgametes are present IMC3 staining is still detectable, most likely adjacent to the membrane of the residual body. The identification of structures stained by the used antibody could be achieved by immune-electron microscopy as used for the characterization of macrogamete development in *E* *. maxima* [40]. This technique still has to be adapted to *I* *. suis* .

In general, little is known about IMC expression in sexual stages so far. Ferguson et al. [41] assume that IMC1 – also a member of the intermediate filament-like IMC proteins – is not expressed in microgamonts of coccidian species. Their conclusion that no IMC is required in ring formation of MORN1 has to be reconsidered because IMC3 expression was not tested in their study. As shown by Anderson-White et al., IMC1 and IMC3 are not necessarily co-expressed [26]. The function of IMC3 in developing microgamonts of *I* *. suis* remains to be elucidated. Moreover, there are no genome data available yet, therefore a comparison with *T. gondii*-sequences of IMC coding genes is not possible at this time point. By using anti-*Tg*IMC3 antibodies it was demonstrated that this tool developed for *T. gondii* is in principal applicable to research on the development of *I* *. suis* . Further antibodies against subcellular structures and proteins of *T. gondii* are currently under evaluation. The next steps to provide the necessary basis for future research with the *in vitro* system are stage-specific proteomics and transcriptomics as well as sequencing of the *I* *. suis* genome as suggested by Clark et al. [8].

With application of established tools from other apicomplexan parasites and possible new tools that still have to be developed for the system presented in this study it might be possible to cover a wide range of topics. This includes the detailed investigation of the epithelial host cell response and mechanisms of pathogenesis in the piglet epithelium, the identification of key factors for the initiation of sexual differentiation, studies on the mechanisms of gametocyte development, or the question if fertilization is necessary for the development of infectious oocysts [42]. Potential new drugs targeting sexual stages could be tested and also mechanisms underlying vaccination strategies based on sexual stage-specific proteins like CoxAbic® [43] might be identified.

## Materials and Methods

### ETHICS STATEMENT

All procedures in this study involving experimental animals were carried out in strict accordance with the recommendations of the Austrian Animal Protection law. The protocols were approved by the Austrian Federal Ministry of Science and Research and the Ethics Committee of the University of Veterinary Medicine Vienna (Permit Numbers: GZ 68.205/78/2005/BrGT; GZ 68.205/95/2007/BrGT; BMWF-68.205/0080-II/3b/2011). All efforts were made to minimize the numbers of animals used to generate *I* *. suis* oocysts and to minimize suffering.

### PARASITES

#### Animals

*I* *. suis* oocysts (strain Wien I) were obtained from experimentally infected suckling piglets. Piglets were raised conventionally with the sow at the animal husbandry facilities of the Institute of Parasitology, University of Veterinary Medicine Vienna, Austria. They were infected with 1000-1500 sporulated oocysts on the fourth day of life. Faecal samples were collected daily on the 7^th^ to 20^th^ day of life and screened for oocysts shedding with autofluorescence microscopy [44] and a modified McMaster-method [45]. When more than 2000 opg were detected faecal samples were used for oocyst isolation.

#### Oocyst isolation

For isolation of oocysts, faeces were suspended in tap water and the 0.25x volume of liquid soap (AC Exakt; E. Mayr Reinigungstechnik, Vösendorf, Austria) and suspended on a magnetic stirrer for 10-15 min. The suspension was filtered through a metal sieve into 50 ml conical tubes to remove solid particles and centrifuged at RT at 2,000 x *g* for 10 min. The supernatant was carefully removed and the pellets were washed twice with tap water (10 min, 2,000 x *g*). Subsequently, pellets were suspended in 25% Percoll® (v/v in tap water, GE Healthcare, Uppsala, Sweden) and centrifuged (10 min, 600 x *g*) to remove most of the lipids. Percoll® traces were removed by an additional washing step with tap water. Pellets were suspended in tap water, poured into a Petri dish and mixed with an equal volume of 4% sodium bichromate (w/v in tap water, ROTH Lactan, Graz, Austria) to reach a final concentration of 2%. For sporulation the oocysts suspensions were aerated twice daily with a pipette and evaporated water was replaced. Sporulated oocysts were transferred to conical 50 ml tubes and stored at 11°C until further use (max. 12 months). Oocysts used for *in vitro* experiments had a mean sporulation rate of 78% (SD 13).

#### Purification of oocysts

Sporulated oocysts were purified in a two-step-procedure modified after Staggs et al. [46], first using a sugar density gradient and then a caesium chloride gradient. Oocyst suspensions were filtered through a mesh (pore size 50 µm) into 50 ml conical tubes and centrifuged for 10 min at 600 x *g*. Pellets were resuspended in 15 ml tap water and mixed with 2.2 M sucrose solution in tap water. This suspension was carefully overlaid with 10 ml tap water and centrifuged for 20 min at 1,200 x *g* without breaks. The upper phase (oocysts in water) was collected and diluted at least 1:3 with tap water. With the remaining sugar solution the steps were repeated after inverting the tube to maximize the oocyst recovery. Diluted oocyst suspensions were centrifuged at 600 x *g* for 10 min without breaks and the supernatant was discarded. All solutions were used at room temperature: The final pellets were either stored in 2% sodium bichromate (w/v) at 11°C or directly used for further purification.

For the second gradient oocysts were resuspended in TE buffer (6.05 g Tris [ROTH Lactan, Graz, Austria] and 3.7 g EDTA [Sigma, Vienna, Austria] in 1000 ml distilled H_2_O, pH 7.2). Four dilutions of CsCl solutions (ROTH Lactan, Graz, Austria) in TE buffer were prepared: stock (21.07 g caesium chloride in 100 ml TE buffer; solution A (20 ml stock and 30 ml TE buffer); solution B (30 ml stock, 20 ml TE buffer and 100 µl 0.5% [w/v] phenol red in tap water); solution C (40 ml stock and 10 ml TE buffer). For the gradient, 10 ml of oocyst suspension in TE buffer was placed in a 50 ml conical tube and slowly underlaid with 8 ml of solution A using a glass pipette, thereafter underlaid with 8 ml of solution B, and finally with 8 ml of solution C. Tubes were centrifuged in a fixed angle rotor at 12,000 x *g* for 60 min at 4°C. Oocysts were recovered from the interphase between solutions A and B. Oocysts from one caesium chloride gradient were distributed to three 50 ml conical tubes and washed twice with tap water (10 min, 600 x *g*) [46]. The final pellet was suspended in 2% sodium bichromate (w/v) for storage at 11°C.

#### Excystation

Excystation methods described for *Eimeria* spp. [47,48] where adapted to *I* *. suis* . Oocysts suspended in 5 ml tap water were treated with sodium hypochlorite (0.24% final concentration of active chloride; ROTH Lactan, Graz, Austria) [21]. After the last washing step oocysts were vortexed with 0.5 mm Precellys® glass beads (Peqlab, Erlangen, Germany) for 15 sec. After transfer of oocysts to a new tube, 5 ml of sterile 0.4% (w/v) pepsin/hydrogen chloride (Sigma, Vienna, Austria; pH adjusted to 3 with 0.1 M hydrogen chloride) were added and incubated for 2 h at 37°C. After centrifugation (10 min, 1000 x *g*) the supernatant was removed and a sterile filtered mixture of 8% fresh porcine bile (v/v, bile not older than 3 weeks, stored at 4°C, obtained from gall bladders of freshly slaughtered pigs) and 0.8% trypsin (w/v, Sigma, Vienna, Austria) in HBSS (PAA, Pasching, Austria) was added. The oocysts where then incubated for 2-3 h under repeated microscopical control. The incubation was terminated when the majority of sporozoites were released. After centrifugation (10 min, 1000 x *g*) the pellet was suspended in DMEM/Ham’s F12 (PAA, Pasching, Austria) with 5% FCS (v/v, PAA, Pasching, Austria) and free sporozoites were counted under trypan blue exclusion. The used method led to mean excystation rates of 63% (SD 25). Considering losses of oocysts during centrifugation a sporozoite recovery rate of 27% (SD 15) in relation to the sporozoites present in the oocysts used for excystation was reached.

### HOST CELLS AND CULTURE CONDITIONS

Intestinal porcine epithelial cells (IPEC-J2, ACC 701, Leibniz Institute DSMZ GmbH, Braunschweig, Germany [24,25]) were maintained in DMEM/Ham’s F12 supplemented with 5% fetal calf serum (FCS), 2 mM L-glutamine, 100 U/ml penicillin, and 0.1 mg/ml streptomycin (PAA, Pasching, Austria). Cells were split at near confluence in a ratio of 1:3 using Accutase^TM^ (PAA, Pasching, Austria) and were cultured at 37°C with 5% CO_2_.

#### Infection

For infection of IPEC-J2 with sporozoites of *I* *. suis* different conditions were used. Medium was supplied with either 5% or 1.25% FCS. Cells were seeded in a density of 4.2 x 10^4^ cells/cm^2^ in flat bottom surface-treated 6-well or 12-well tissue culture plates or 25 cm^2^ cell culture flasks (PAA, Pasching, Austria). Cells were infected two days after seeding with sporozoites of *I* *. suis* suspended in culture medium in ratios of sporozoites: cells of 1:5, 1:10, 1:40, 1:50, 1:100, 1:200, 1: 400 or 1: 500, respectively. For quantification cells were seeded in 8-well tissue culture treated µ-slides (chamber slides for live microscopy) suitable for differential interference contrast (DIC) microscopy with oil immersion (Ibidi, Martinsried, Germany). Infected cells were incubated at 37°C and 5% CO_2_.

#### Oocyst harvest and sporulation

For isolation of oocysts from cell culture, supernatants were removed from infected cell cultures and centrifuged at 600 x *g* for 10 min. The pellet was then resuspended in tap water and transferred to 6-well cell culture plates for sporulation. Oocyst suspensions were kept at room temperature in the dark and were aerated daily as described for oocysts isolation from porcine faeces. Sporulation was controlled by light microscopy. The number of oocysts and sporulation rates were assessed by counting all oocysts per well. The recovery rate was calculated as follows: 100/(used sporozoites/8) x harvested oocysts. The quantity of sporozoites was divided by 8 to calculate the number of successfully excysted oocysts necessary for this amount.

### SEMI-QUANTITATIVE EVALUATION

For semi-quantitative evaluation infected cells were examined with phase contrast microscopy on dpi 2, 5, 7, 9, 12, and 15 with an Olympus IX71 inverted microscope (Olympus Austria GmbH, Vienna, Austria). A score was used for evaluation of cell condition reflecting the integrity of the cell monolayer and parasites as follows: for host cells 0 (no cells left), 0.5 (only 10% or less cells intact), 1 (40% of cells intact), 2 (60-80% of cell intact), 3 (cell monolayer intact); for parasites (cells infected with merozoites, free merozoites, gametes, or non-sporulated oocysts, respectively): 0 (no stages), 0.5 (at least single parasites seen per well), 1 (stages seen but only few per well), 2 (many stages found, only single stages per field of vision), 3 (many per field of vision).

### QUANTITATIVE EVALUATION

Pictures from 30 fields of vision were taken per well of 8-well µ-slides at different dpi with DIC using an Olympus IX71 inverted microscope, an 60x oil immersion objective, and a ColorView III camera (Olympus Austria GmbH, Vienna, Austria). For maintaining optimum culture conditions during microscopy a stage top incubator for life cell imaging (Ibidi, Martinsried, Germany) with 37°C and 5% CO_2_ was used. Cells and parasitic stages were counted using Cell^F^ imaging software (Olympus Soft Imaging Solutions GmbH, Münster, Germany); the length of intracellular zoites was measured using the same program.

### ELECTRON MICROSCOPY

For imaging of the parasite morphology, infected cells were harvested on dpi 5, 7, 8, 12, and 13 with Accutase^TM^ (PAA, Pasching, Austria). The harvested cells were fixed in 3% glutaraldehyde, washed in Sörensen’s phosphate buffer and embedded in a 1.5% agar gel prepared in Sörensen’s buffer. After setting of the gel, the cell pellet was post-fixed in 1% osmium tetroxide in Sörensen’s buffer and washed again in Sörensen’s buffer. After dehydration in alcohols of increasing concentrations, the samples were infiltrated with propylene oxide, followed by increasing ratios of epoxy resin-propylene oxide and finally pure resin. After an additional change, the resin was polymerized at 60°C. Ultrathin sections were contrasted with lead citrate and uranyl acetate and examined with an EM 900 electron microscope (Zeiss, Oberkochen, Germany).

### IMC3 STAINING

For staining of the inner membrane complex, infected cells cultured on glass cover slips were fixed with 100% methanol and blocked with 4% BSA (Sigma-Aldrich, Vienna, Austria) in PBS. The primary anti-IMC3 rat polyclonal antibody was a kind gift of Marc-Jan Gubbels, Boston College [49]. As secondary antibody, goat anti-rat-A488 (Invitrogen, Eugene, OR, USA) was used. DNA was visualized by staining with 4,6-diamidino-2-phenylindole (DAPI) for 2 min. The samples were examined with a confocal microscope (Zeiss LSM 510 Meta/Axiovert 200M) using BP 420-480 Filters (Ex 405 MT 405 nm : 10.0%, Ex 488 MT 488 nm : 5.0%). Pictures were analyzed with ZEN 2009 Light Edition (Carl Zeiss Microimaging GmbH, Jena, Germany).

### STATISTICAL ANALYSIS

Statistical evaluation was performed using PASW Statistics 17.0 (SPSS Inc., Chicago, IL, USA). The influence of the FCS concentration in the culture media on the development of parasitic stages and the host cell condition was analyzed by comparing 1.25% FCS with 5% FCS concentration using Mann-Whitney (Wilcoxon) two sample tests; significance was assumed for *p* ≤ 0.05; for this analyses data were split by infection dose and dpi.

The influence of the infection dose (host cells: sporozoites = 1:10, 1:100, 1:200, 1:400) on parasite development and host cells condition was analyzed for the optimal FCS concentration in culture medium of 5% by ANOVA investigating the main effect infection dose, not normally distributed data were analyzed with Kruskal-Wallis tests. Significance was assumed for *p* ≤ 0.05. Post-hoc multiple comparisons with Bonferroni corrections were computed to identify differences between all infection doses in case of normally distributed data, significance was assumed for *p* ≤ 0.05. Alternatively, multiple comparisons were computed with Mann-Whitney (Wilcoxon) two sample tests for not normally distributed data. The confidence intervals after multiple comparisons of these data were corrected according to Bonferroni. Consequently, significance was assumed for *p* ≤ 0.008 for results from Mann-Whitney (Wilcoxon) two sample tests used for multiple comparisons. Only significant results were tabulated for these analyses.

## Supporting Information

Figure S1 Development of *I* *. suis* merozoites in IPEC-J2 culture comparing 5% (grey) and 1.25% (white) fetal calf serum. Results of semi-quantitative evaluation of intra- and extracellular merozoites are shown; presence of parasitic stages was scored from 0 (negative) to 3 (many/field of vision). Host cells were infected with a ratio of sporozoites: cells of 1:10, 1:100, 1:200, and 1:400, respectively. Significant differences found by Mann-Whitney (Wilcoxon) two-sample tests comparing medium conditions are indicated by red asterisks (*p ≤ 0.05, **p ≤ 0.01); each box plot spans the interquartile range of on the y-axis, with the inside line indicating the median; whiskers extend to the minimum and maximum values excluding outside and far out values represented by circles or black asterisks; dpi, days post infection.
(TIF)

Figure S2 Development of *I* *. suis* gametocytes and oocysts in IPEC-J2 culture and host cell condition comparing 5% (grey) and 1.25% (white) fetal calf serum. Results of semi-quantitative evaluation of intracellular gametocytes, oocysts and host cell condition are shown; presence of parasitic stages was scored from 0 (negative) to 3 (many/field of vision), cell condition was scored from 0 (no cells left) to 3 (cell monolayer intact). Host cells were infected with a ratio of sporozoites: cells of 1:10, 1:100, 1:200, and 1:400, respectively. Significant differences found by Mann-Whitney (Wilcoxon) two-sample tests comparing medium conditions are indicated by red asterisks (*p ≤ 0.05, **p ≤ 0.01); each box plot spans the interquartile range of on the y-axis, with the inside line indicating the median; whiskers extend to the minimum and maximum values excluding outside and far out values represented by circles or black asterisks; dpi, days post infection.
(TIF)

Figure S3 Sporulated oocysts of *I* *. suis* contaminating the infection material after 9 days in culture. (A) degraded oocyst with granular content in the sporocyst but intact walls attached to or within a host cell (IPEC-J2); (B) degraded oocysts with collapsed oocyst and sporocyst walls attached to or within a host cell; bar = 20 µm.
(TIF)

Table S1 Significant influence of the infection dose on parasite development and cell condition over time in culture medium with 5% FCS. (PDF)
