# Supplementary figures and images for: Isospora suis in an Epithelial Cell Culture System – An In Vitro Model for Sexual Development in Coccidia
Source: PLoS One. 2013 Jul 5;8(7):e69797. doi: 10.1371/journal.pone.0069797 (PMC3702598; doi:10.1371/journal.pone.0069797)

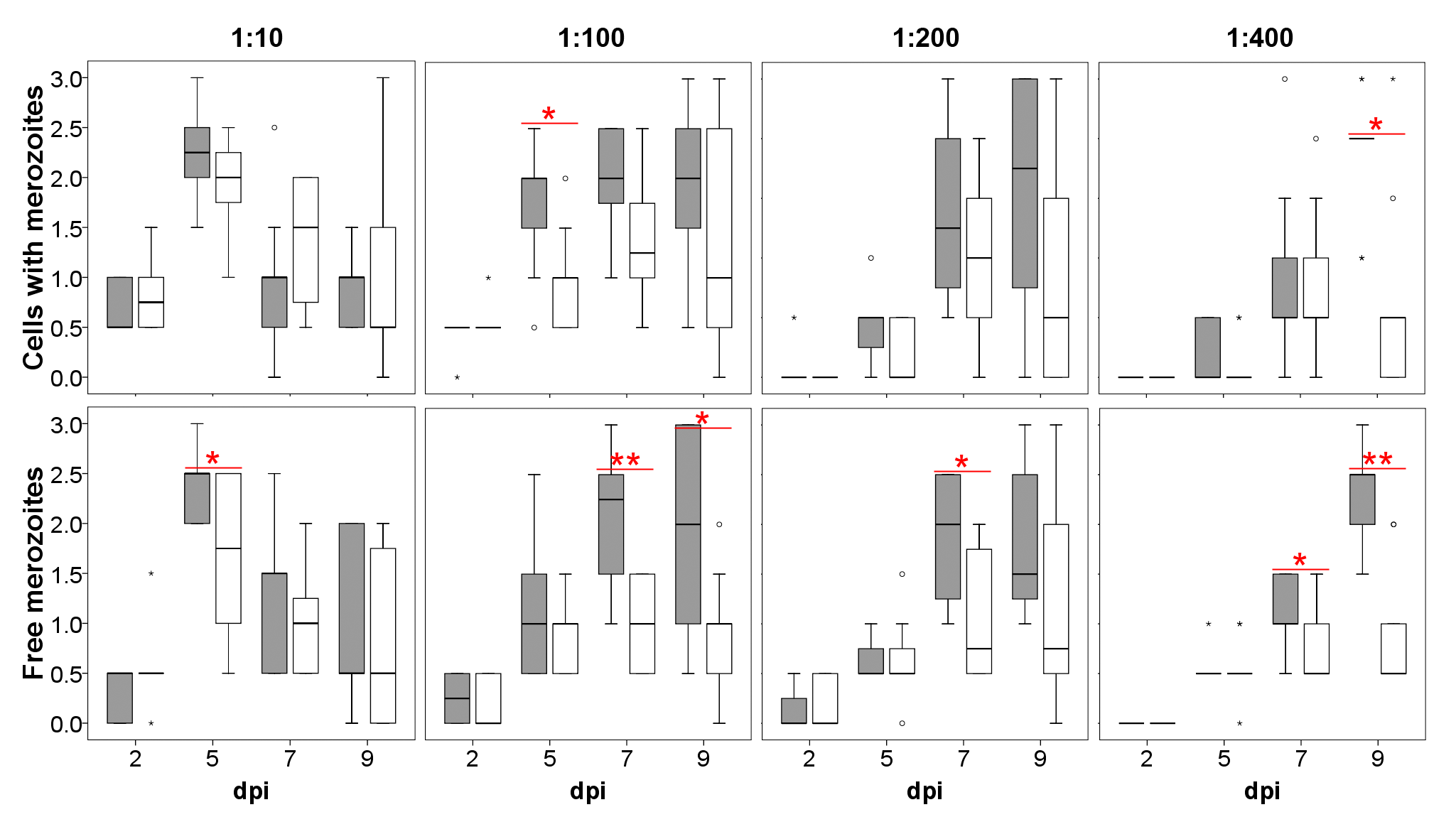

Supplement: Figure S1 — Results of semi-quantitative evaluation of intra- and extracellular merozoites are shown; presence of parasitic stages was scored from 0 (negative) to 3 (many/field of vision). Host cells were infected with a ratio of sporozoites: cells of 1:10, 1:100, 1:200, and 1:400, respectively. Significant differences found by Mann-Whitney (Wilcoxon) two-sample tests comparing medium conditions are indicated by red asterisks (*p ≤ 0.05, **p ≤ 0.01); each box plot spans the interquartile range of on the y-axis, with the inside line indicating the median; whiskers extend to the minimum and maximum values excluding outside and far out values represented by circles or black asterisks; dpi, days post infection. (TIF) [file pone.0069797.s001.tif]

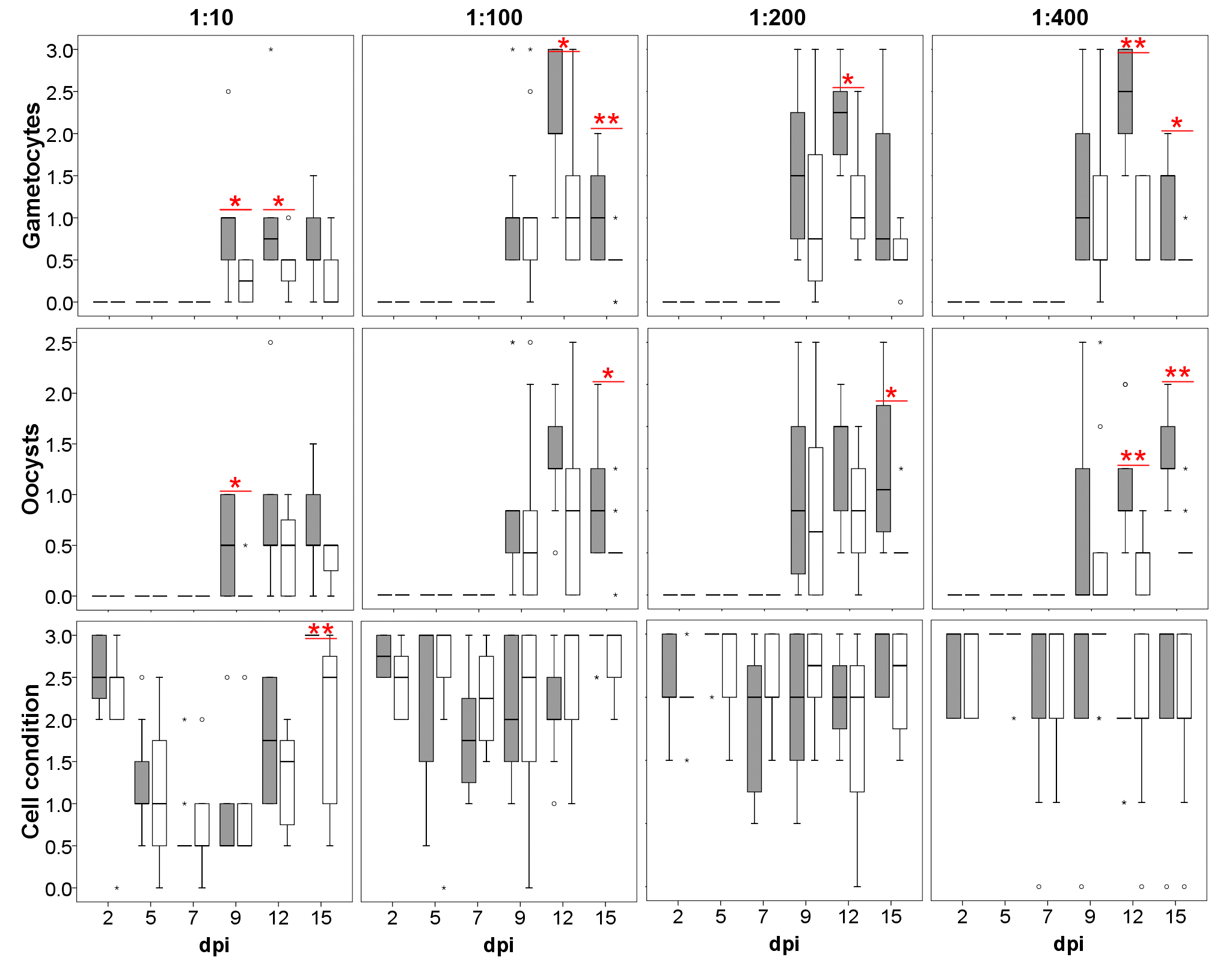

Supplement: Figure S2 — Results of semi-quantitative evaluation of intracellular gametocytes, oocysts and host cell condition are shown; presence of parasitic stages was scored from 0 (negative) to 3 (many/field of vision), cell condition was scored from 0 (no cells left) to 3 (cell monolayer intact). Host cells were infected with a ratio of sporozoites: cells of 1:10, 1:100, 1:200, and 1:400, respectively. Significant differences found by Mann-Whitney (Wilcoxon) two-sample tests comparing medium conditions are indicated by red asterisks (*p ≤ 0.05, **p ≤ 0.01); each box plot spans the interquartile range of on the y-axis, with the inside line indicating the median; whiskers extend to the minimum and maximum values excluding outside and far out values represented by circles or black asterisks; dpi, days post infection. (TIF) [file pone.0069797.s002.tif]

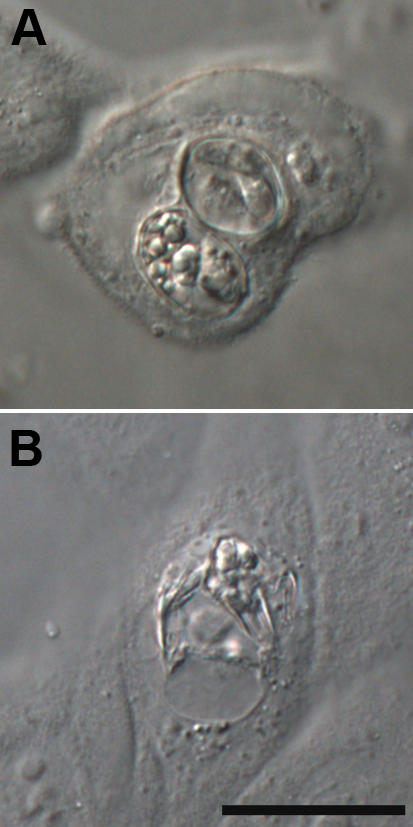

Supplement: Figure S3 — (A) degraded oocyst with granular content in the sporocyst but intact walls attached to or within a host cell (IPEC-J2); (B) degraded oocysts with collapsed oocyst and sporocyst walls attached to or within a host cell; bar = 20 µm. (TIF) [file pone.0069797.s003.tif]
