# Supplementary material for: Isospora suis in an Epithelial Cell Culture System – An In Vitro Model for Sexual Development in Coccidia
Source: PLoS One. 2013 Jul 5;8(7):e69797. doi: 10.1371/journal.pone.0069797 (PMC3702598; doi:10.1371/journal.pone.0069797)
Supplement: Table S1 — (PDF) [file pone.0069797.s004.pdf]

Table S1 Significant influence of the infection dose on parasite development and cell condition over time in culture medium with 5% FCS.

	dpi	*p*-value	df	F	Chi-square
**Cells with merozoites**	5	<0.001	3, 32	24.5	n.a.
	7	0.018	3, 30	3.9	n.a.
	9	<0.001	3, 32	8.9	n.a.
**Free merozoites**	2	0.027	3, 29	3.5	n.a.
	5	0.001§	3	n.a.	25.4
	9	0.005	3, 32	5.2	n.a.
**Gametocytes**	12	<0.001	3, 32	8.7	n.a.
**Oocysts**	12	0.004	3, 32	3.0	n.a.
	15	0.049	3, 31	2.9	n.a.
**Cell condition**	5	<0.001§	3	n.a.	21.8
	7	<0.001	3, 30	17.7	n.a.
	9	<0.001	3, 32	15.3	n.a.
	12	0.033§	3	n.a.	8.7

P-values from ANOVA comparing four different infection doses (sporozoites:cells = 1:10, 1:100, 1:200, 1:400) are shown. §data were not normally distributed, the *p*-values from Kruskal-Wallis tests are shown; dpi: days post infection; df: degrees of freedom; n.a.: not applicable because of used statistical test; significance was assumed for $p \leq 0.05$.
